# The Effect of the Use of Microencapsulated Stabilizers on Textural Rheological and Physicochemical Properties of Ice Cream

**DOI:** 10.1002/fsn3.71570

**Published:** 2026-03-03

**Authors:** Mehmet Kilinc, Gülden İlkilinc

**Affiliations:** ^1^ Department of Food Engineering, Faculty of Engineering Afyon Kocatepe University Afyonkarahisar Türkiye

**Keywords:** coating, ice cream, quality, stabilizer, texture

## Abstract

This study investigated the effects of microencapsulated stabilizers guar gum, locust bean gum, xanthan gum, and carboxymethyl cellulose (CMC) on the physicochemical, rheological, melting, textural and sensory properties of ice cream. All stabilizers were coated onto an iodine carrageenan matrix, which served as a reinforced encapsulation wall material to enhance microcapsule integrity. SEM analysis revealed smoother and more compact microcapsules, improving stabilizer dispersion and interaction within the ice cream matrix. Microencapsulation produced statistically significant improvements (*p* < 0.05) in rheological behavior. Guar and xanthan gum treatments exhibited markedly higher G′, G″, and G* values—up to 35%–40% greater than the control—indicating stronger viscoelastic networks and enhanced water immobilization. Melting resistance also increased significantly (*p* < 0.05); locust bean gum extended first drip time by over 25%, while xanthan gum provided the greatest structural endurance during thawing. Overrun and textural parameters were similarly influenced: CMC and xanthan improved aeration efficiency, whereas guar gum generated a firmer and more cohesive structure due to its higher viscosity. Sensory evaluation confirmed these technological enhancements. Panelists rated the microencapsulated formulations significantly higher (*p* < 0.05) in texture, melting resistance, and mouthfeel, with guar gum achieving the highest overall acceptability. Importantly, no off‐flavors were detected, demonstrating the sensory compatibility of the encapsulation system. Overall, coating stabilizers onto an iodine carrageenan matrix significantly enhanced their functional performance, improved freeze–thaw behavior, reduced ice recrystallization, and elevated sensory quality. These findings highlight microencapsulation as a powerful strategy for developing next‐generation ice cream formulations with superior structural stability and consumer appeal.

## Graphical abstract

Microencapsulation of stabilizers using an iodine–carrageenan matrix significantly enhanced the structural and functional properties of ice cream. Encapsulated guar gum, locust bean gum, xanthan gum, and CMC improved rheological behavior, melting resistance, texture, and microstructural stability compared to the control. This approach effectively reduced ice recrystallization and enhanced sensory acceptance, demonstrating the potential of microencapsulated stabilizers for high‐quality ice cream production.

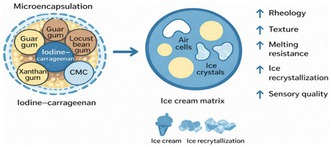

## 1 Introduction

Ice cream is a complex multiphase frozen dairy product whose quality depends on the interactions among air cells, fat globules, ice crystals, and the unfrozen serum phase. These structural components determine key physicochemical and sensory attributes such as smoothness, meltdown behavior, and textural stability (Goff and Hartel 2013). One of the primary challenges in ice cream production is controlling ice recrystallization during storage. Temperature fluctuations cause small ice crystals to melt and grow into larger ones, leading to coarse texture and loss of sensory quality (Flores and Goff 2020; Shevade and Hartel 2022).

Hydrocolloid stabilizers are widely used to control water mobility, delay melting, improve overrun, and enhance viscosity. Stabilizers such as guar gum, xanthan gum, locust bean gum, and carboxymethyl cellulose (CMC) have specific functional roles based on their molecular structure and hydration mechanisms (Acı and Özcan 2007; Tekinşen 1997). Guar gum is known for its high water‐binding capacity and strong thickening effect, while xanthan gum contributes to freeze–thaw stability and shear‐thinning behavior. CMC improves foam stability and overrun, whereas locust bean gum enhances body and smoothness through synergistic gelation interactions. Recent studies also emphasize that hydrocolloid blends significantly influence melting rate, hardness, and structural collapse in dairy and plant‐based ice creams (Rahman et al. 2025; Raisel et al. 2024).

However, the efficacy of directly adding these stabilizers to the ice cream mix can be limited. Premature hydration or non‐uniform dispersion can lead to suboptimal functionality. Furthermore, the effectiveness of stabilizers can diminish over the product's shelf life, failing to provide long‐term protection against recrystallization. To overcome these limitations, innovative processing technologies such as microencapsulation are studied as a way to enhance ingredient performance (Gharsallaoui et al. 2007). Microencapsulation is a process in which active ingredients are coated with a protective material, creating micro‐sized capsules. This technology offers many advantages, including the protection of sensitive ingredients from adverse environmental conditions, controlled release at a specific time or location, and improved handling of functional ingredients (Anandharamakrishnan and Parthasarathi 2019; Augustin and Sanguansri 2020). The microencapsulation process involves the encapsulation of active ingredients in micro‐sized capsules to protect them from external factors and release in a controlled manner. In the food industry, this technology is utilized to enhance the efficacy of stabilizers, preserve sensory characteristics, and achieve regulated release of ingredients in specific processes (Fang and Bhandari 2010; Anandharamakrishnan and Parthasarathi 2019).

In the context of ice cream production, microencapsulating stabilizers offer a strategic advantage. By creating a protective barrier around the stabilizer, its interaction with water can be delayed until the critical freezing and storage stages, where its function is most needed to inhibit ice crystal growth. This controlled‐release mechanism can lead to more effective and prolonged stabilization, resulting in superior structural quality and an extended shelf life for the final product (Shevade and Hartel 2022). The application of microencapsulation can therefore provide a more robust solution for maintaining the desired smooth texture of ice cream over time.

However, the functional performance of stabilizers can diminish due to premature hydration, uneven dispersion, or degradation during frozen storage. Microencapsulation has emerged as a promising approach to improve ingredient stability and functionality. By surrounding active materials with protective wall systems, microencapsulation allows controlled release, improved solubility, and enhanced resistance to environmental stress (Gharsallaoui et al. 2007; Anandharamakrishnan and Parthasarathi 2019). Traditionally, microencapsulation has been applied to probiotics, vitamins, and flavors, but recent research highlights its potential for improving the physicochemical stability of frozen dairy products (Junaid et al. 2024).

Although microencapsulation has been extensively applied to bioactive compounds, its use on ice cream stabilizers is novel. Iodine carrageenan was selected as the core encapsulation matrix due to its strong interaction with milk proteins, high gel strength, freeze–thaw stability, and ability to provide a controlled hydration and release platform for different stabilizer coatings in ice cream systems. This study introduces a new technological strategy in ice cream formulation by microencapsulating iodine carrageenan with different coating materials (xanthan gum, guar gum, locust bean gum, and CMC). This approach enables controlled hydration of stabilizers during freezing and storage, potentially enhancing rheological behavior, melting resistance, and structural uniformity. To the best of our knowledge, no previous study has systematically compared microencapsulated stabilizer systems in ice cream production.

This research evaluates the effects of microencapsulated stabilizers on ice cream quality parameters including dry matter, pH, titratable acidity, color (L*, a*, b*), melting behavior, overrun, hardness, and rheological properties. Additionally, SEM was used to analyze the surface morphology of microcapsules. The findings are expected to support industrial applications for producing ice creams with improved stability, texture, and structural integrity.

## 2 Materials and Methods

### 2.1 Materials

Raw cow's milk, granulated sugar, and cream (35% fat, President, France) were purchased from local suppliers in Afyonkarahisar, Türkiye. Skim milk powder (SMP; Bağdat Gıda, Türkiye) and food‐grade emulsifier mono‐ and diglycerides (E471; Puratos, Belgium) were used in the ice cream mix. The primary stabilizer was iodine carrageenan (Sigma‐Aldrich, USA). Coating materials used for microencapsulation were guar gum (Naturex, France), locust bean gum (carob gum; Nexira, France), xanthan gum (CP Kelco, USA), and carboxymethyl cellulose (CMC; Sigma‐Aldrich, USA). All chemicals used in analytical procedures were analytical grade. The ice cream formulation is presented in Table 1.

**TABLE 1:** Ice cream formulation.

Ingredient (%)	Control iodine (I)	Guar gum–coated iodine (S1I)	Locust bean gum–coated iodine (S2I)	Xanthan gum–coated iodine (XI)	CMC–coated iodine (S3I)
Milk	72.2	72.2	72.2	72.2	72.2
Sucrose	17.0	17.0	17.0	17.0	17.0
Cream	5.0	5.0	5.0	5.0	5.0
Skim milk powder	5.0	5.0	5.0	5.0	5.0
Emulsifier (E471)	0.3	0.3	0.3	0.3	0.3
Free stabilizer	0.5	—	—	—	—
Microencapsulated stabilizer	—	0.5	0.5	0.5	0.5

### 2.2 Methods

#### 2.2.1 Preparation of Microencapsulated Stabilizers

Microencapsulation was performed using an emulsion–microfluidization–freeze‐drying method. First, an aqueous phase was prepared by dissolving iodine carrageenan (1%, w/v) and one of the coating polymers (xanthan gum, guar gum, locust bean gum, or CMC at 0.5%, w/v) in deionized water at 60°C with continuous stirring (IKA RW20, Germany). A separate oil phase (sunflower oil, 5%) was then added slowly to the aqueous phase and homogenized using a high‐speed homogenizer (Ultra‐Turrax T25, IKA, Germany) at 10,000 rpm for 3 min. The pre‐emulsion was passed through a high‐pressure microfluidizer (Microfluidics M‐110P, USA) at 100 MPa, with three consecutive cycles, to obtain nanoscale droplets. This ensured uniform coating distribution around the carrageenan core. The resulting microcapsules were freeze‐dried using a laboratory lyophilizer (Christ Alpha 1–2 LDplus, Germany) under −48°C and 0.050 mbar for 48 h. The dried powders were stored at −20°C in airtight containers until use.

#### 2.2.2 Ice Cream Production

**FIGURE 1:**
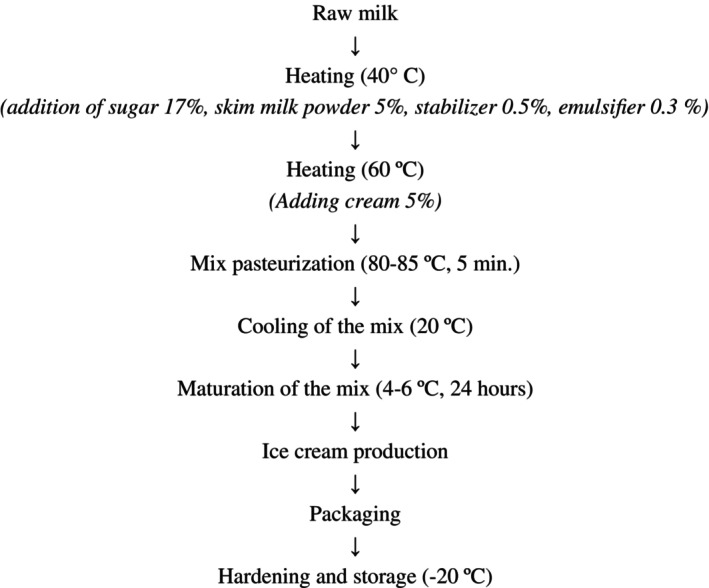
Ice cream production flow chart (Kılınc and Sevik 2021).

Ice cream mixes were prepared by combining the specified ingredients according to the production flowchart presented in Figure 1. The process was initiated by heating raw milk to 40°C, at which point sugar, skim milk powder, the prepared microencapsulated stabilizer, and the emulsifier were added. The mix was further heated to 60°C, and the cream was added with continuous stirring.

The entire mixture was then pasteurized at 85°C for 5 min. Then, the ice cream mix was cooled to 20°C and transferred to containers for maturation (aging) at 4°C for 24 h. After maturation, the mix was frozen in a batch freezer (CRM, Gel 25 C, Italy). The ice cream was then packaged and transferred to a hardening freezer for storage at −20°C for 3 months.

#### 2.2.3 Scanning Electron Microscopy (SEM)

Samples were examined using an FEI QUANTA FEG 250 model field‐emission scanning electron microscope (FE‐SEM) without any prior pretreatment. The electron microscopy imaging was performed using a large‐field low‐vacuum secondary electron detector (LFD). Samples were mounted on specially designed sample holders (approximately 1.0 cm in diameter) using carbon conductive double‐sided tape, and imaging was carried out under standard laboratory conditions. SEM observations were conducted within a magnification range of 1000× – 8000×, using an accelerating voltage of 2–10 kV and a working distance of 8–12 mm, which provided optimal image resolution and surface detail. These parameters were selected to ensure accurate visualization of particle morphology, surface roughness, and structural differences among samples.

#### 2.2.4 Texture Analysis of Ice Cream Mix

The firmness, consistency, cohesiveness, and index of viscosity of the ice cream mix were determined using a TA.XT Plus Texture Analyzer (Stable Micro Systems, Godalming, Surrey, UK) equipped with a back extrusion rig according to the method described by Sert et al. (2017).

#### 2.2.5 Dry Matter Content

The drying containers were cleaned and placed in drying cabinets (Thermo Scientific Heraeus) with the lids partially open at 103°C until a constant weight was achieved. After removal, containers were cooled in a desiccator and weighed to obtain the drying tare (empty dried container weight). An ice cream sample weighing 5–6 g was placed into each drying container. Containers were then placed in an oven at 105°C for 1.5–2 h. After drying, the lids were closed, and the containers were cooled in a desiccator and weighed. The dry matter content was calculated using Equation 1 (AOAC International 2019).

(1) $$\%\mathrm{Dry}\;\text{Matter}=\frac{\;\text{final weight}-\text{Drying tare}}{\text{Sample amount}\;}x100$$

#### 2.2.6 Degree of Acidity (pH) Measurement

After 100 g of the ice cream sample was melted in a container to 20°C and washed with distilled water, the pH meter electrode was placed in the sample, and the reading was allowed to stabilize before the measurement was taken.

#### 2.2.7 Titratable Acidity

9 g of the ice cream sample were mixed with 9 mL of distilled water at room temperature together with 1% phenolphthalein indicator. The samples were titrated with 0.1 N NaOH until a permanent light pink color was observed. The titratable acidity was expressed as % lactic acid using the following formula (Akalın et al. 2018) Equation 2,

(2) $$\mathrm{LA}\%=\frac{\mathrm{Nx}0.009\mathrm{xV}}{m}x100$$

**N:** Normality of NaOH solution.

**V:** Volume of 0.1 N NaOH used in titration (mL).

**m:** Weight of the ice cream sample (g).

#### 2.2.8 Color Measurement

The color values of the freezing and coating powder samples were measured using an automatic colorimeter (Hunter Lab colorimeter). First, the instrument was calibrated using a standard calibration scale. The ice cream and powder samples were then filled into the cuvette of the device and L*, a* and b* values were determined (Çelik et al. 2009). The total color difference (ΔE*) was calculated based on the CIELab color system using L*, a*, and b* values, with all ΔE* values determined relative to the control sample.

#### 2.2.9 First Drip Time

The first drip time was measured using 400 mL beakers and wire screens with a wire thickness of approximately 1.0 mm and about 10 holes per 2.50 cm. First, the tare weights of the beakers and wire grids were recorded. Approximately 20 g of ice cream stored at −25°C was then placed on the wire grids. The samples were kept at 20°C, and the time until the first drop of melted ice cream passed through the grid was measured in seconds (Kılınc and Sevik 2021).

#### 2.2.10 Complete Melting Time

The hardened ice cream samples were allowed to melt on a 0.2 cm wire mesh screen placed over a beaker at 20°C, and the time required for complete melting was recorded in minutes (Kılınc and Sevik 2021). This procedure is a widely adopted approach for evaluating the melt‐down characteristics of ice cream products.

#### 2.2.11 Overrun Determination

A specific volume of ice cream was transferred to a calibrated cylinder, ensuring that there were no voids, and then weighed. The same ice cream sample was then placed in a beaker and melted in a water bath. Subsequently, the molten mix, maintaining the same volume, was transferred back to the pre‐cleaned and measured cylinder, and a second weighing was performed by following the principles outlined by (Kılınc and Sevik 2021). The overrun percentage was calculated by using the following formula: Equation 3

(3) $$\text{Overrun}\;\left(\%\right)=\frac{\text{Weight of}\;\mathrm{mix}-\text{Weight of}\;\mathrm{ice}\;\text{cream}}{\text{Weight of}\;\mathrm{ice}\;\text{cream}}x100$$

#### 2.2.12 Textural Properties (Hardness)

The hardness of the frozen samples was determined using a TA.XT Plus Texture Analyzer (Stable Micro Systems, Part Code: P/5) equipped with a 5 mm diameter cylindrical stainless‐steel probe. Before the measurement, the samples were tempered at −15°C for 24 h. After the specified tempering period, three measurements were taken from three separate containers for each sample, and the average of these measurements was calculated, as described by Akalın et al. (2008).

#### 2.2.13 Rheological Properties

In oscillation tests, samples were subjected to a sinusoidal oscillatory stress or strain, and the storage modulus (G′) and loss modulus (G′′) values were determined against specific frequency values. The overall response of the samples to sinusoidal stress is characterized by the complex modulus (G*) and complex viscosity (η*), which are calculated from the storage (G′) and loss (G′′) moduli, as described by Yılmaz (2014).

$$G^{*}={\left[{\left(G^{\prime }\right)}^{2}+{\left(G^{\prime \prime }\right)}^{2}\right]}^{1/2}$$

$$\eta^{*}=G^{*}/\omega \;$$

#### 2.2.14 Sensory Analysis

Sensory evaluation was conducted with 12 trained panelists following ISO 8586 procedures. Microencapsulated‐stabilizer ice cream samples were tempered to −12°C ± 1°C and served in randomized, coded cups under controlled sensory booths. Panelists assessed appearance/color, aroma, flavor, texture–mouthfeel, melting resistance, off‐flavor, and overall acceptability using a 9‐point hedonic scale (ISO 8586:2012 n.d.).

### 2.3 Statistical Analysis

Statistical analyses were conducted utilizing SPSS 22.0 (SPSS Inc., New York, USA). Statistically significant differences between mean values were determined using Tukey's multiple comparison tests at a confidence interval of *p* < 0.05. Results are presented as mean ± standard deviation.

## 3 Result and Disscusion

**FIGURE 2:**
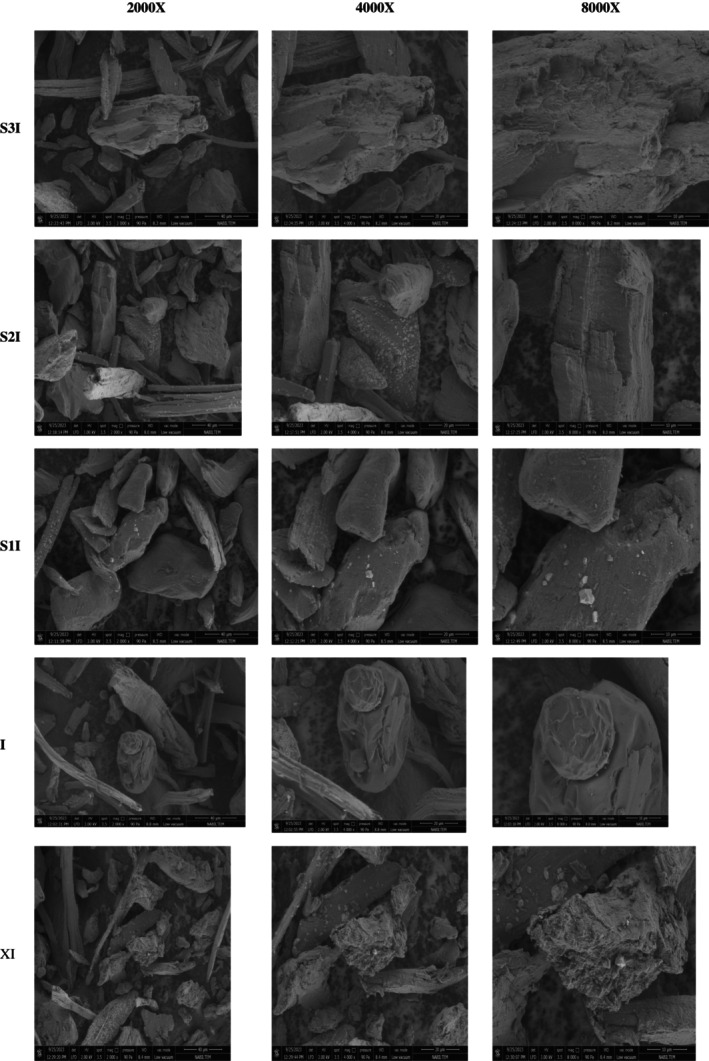
SEM images of the coating samples.

SEM images of the coating samples are presented in Figure 2. Surface smoothness, cracks or irregularities are observed in the uncoated form in iodine. Capsules coated with xanthan gum show a certain regularity and homogeneous distribution between the capsules due to the high water binding capacity of xanthan gum. It was reported in the literature that capsules coated with xanthan gum have a smooth and homogeneous structure (Flores and Goff 2020). Xanthan gum increases the stability of the capsules thanks to its viscous structure. At high magnifications, the capsules can be seen to have a smooth structure without cracks on the surface. As reported in the literature, particles microencapsulated with xanthan gum usually form regular and stable capsules (Goff and Hartel 2013).

CMC was demonstrated to form a thin and homogeneous layer on the capsules, resulting in a uniform distribution. CMC was shown to provide a coating on the capsules that is both homogeneous and smooth (Zhang et al. 2015).

In the context of microencapsulation processes, locust bean gum typically results in the formation of a rougher structure. This increased roughness is discernible on the surface of the capsules. This phenomenon is attributable to the inherent viscosity and structural characteristics of locust bean. The locust bean coating was shown to form a rougher and more complex surface structure in comparison to other stabilizers (Mezger 2018). Distributions deviating from uniformity, in conjunction with surfaces exhibiting a lack of smoothness, were identified within the capsules.

Guar gum was reported to yield regular and smooth surfaces in microencapsulation processes. Despite the presence of irregularities or cracks on the surface of the capsules coated with guar gum, it was determined that the surface remained smooth in general. Reineccius (2004) reported that the microencapsulation process with guar gum typically resulted in the production of smooth surfaces.

SEM analysis confirmed that microencapsulated stabilizers generate a more compact, homogeneous, and structurally resilient matrix, while non‐encapsulated or insufficient stabilizer systems result in weaker, porous structures prone to collapse. This improved microstructural organization explains the superior physicochemical and melting properties observed in microencapsulated formulations.

### 3.1 Textural Properties of Ice Cream Mix

The textural properties of the ice cream mixtures produced during the ice cream production process are given in Table 2. The highest hardness values of ice cream samples were found in control iodine samples (89.67g) and the lowest hardness values in CMC iodine samples (41.89g). The highest consistency values of ice cream samples were found in control samples (1274.74g) and the lowest consistency values in CMC iodine samples (690.69g). The highest cohesion values of ice cream samples were found in control samples with 63.44g and the lowest cohesion values in carboxymethyl cellulose iodate samples (36.94g). The highest index viscosity values of ice cream samples were found in control samples (119.65g.s) and the lowest index viscosity values in carboxymethyl cellulose iodate samples (75.11g.s). Different letters in the same column indicate a statistical difference between the data (*p* < 0.05).

**TABLE 2:** Textural properties of ice cream mix.

	Firmness (g)	Consistency (g.s.)	Cohesiveness (g)	Index of viscosity (g.s)
I	89.67 ± 10.14^a^	1274.74 ± 36.26^a^	−63.44 ± 7.81^e^	−119.65 ± 13.53^dc^
S1I	48.64 ± 5.15^cd^	795.22 ± 95.20^bc^	−40.12 ± 1.99^b^	−82.77 ± 6.69^ba^
S2I	60.52 ± 5.75^b^	929.79 ± 96.54^b^	−49.00 ± 0.47^d^	−105.79 ± 2.32^c^
XI	50.37 ± 0.39^c^	810.45 ± 33.88^b^	−44.20 ± 0.55^c^	−86.45 ± 0.96^b^
S3I	41.89 ± 0.86^d^	690.69 ± 35.78^c^	−36.94 ± 0.69^a^	−75.11 ± 0.60^a^

*Note:* Different letters in the same column indicate a statistically significant difference between the data (*p* < 0.05). ± = Standard deviation of three repetitions.

The sample coated with carrageenan showed significantly higher firmness compared to formulations coated with other stabilizers (*p* < 0.05). The formulation in iodine showed significantly higher consistency compared to all other stabilizers (*p* < 0.05). Consistency differences were observed between formulations coated with locust bean gum, xanthan gum, and guar gum, whereas no statistically significant difference was observed between guar gum and xanthan gum. Iodine yielded the highest stickiness value, while the CMC‐coated formulation showed the lowest value. İodine coated with guar gum showed higher adhesion than CMC and this difference was significant (*p* < 0.05) In terms of viscosity, significant differences were observed between the iodine–carrageenan–based encapsulation matrix and the CMC‐coated formulation as well as the other stabilizer‐coated samples (*p* < 0.05). The iodine–carrageenan matrix exhibited the highest viscosity index, while locust bean gum showed the highest viscosity among the coated stabilizer formulations.

According to Goff and Hartel (2013), carrageenan maintains stability, especially in dairy products, and improves product quality by inhibiting the growth of ice crystals. Furthermore, the high viscosity index indicates that carrageenan provides a dense structure in the ice cream mix. Literature supports these results.

Guar gum provides a softer and smoother texture and has lower firmness and consistency values than carrageenan and carob gum. Reineccius (2004) reported that guar gum provides low viscosity and stiffness and emphasized that this stabilizer is suitable for creamier and softer products.

Locust bean gum offers lower stiffness and consistency values compared to carrageenan but still provides high stickiness and viscosity. Mezger (2018) reported that locust bean gum increases consistency and density in food products, particularly thanks to its binding properties, and that this stabilizer increases viscosity. This finding demonstrates the ability of carob gum to impart a dense structure in microencapsulation.

Flores and Goff (2020) reported that xanthan gum increases viscosity in ice cream production and ensures stability even at low temperatures. The intermediate results of xanthan gum in consistency and viscosity index are consistent with the properties mentioned in the literature.

CMC has the lowest hardness and consistency values. Zhang et al. (2015) reported that CMC was effective as an emulsion stabilizer but provided lower consistency compared to carrageenan and locust bean gum. CMC creates less sticky and less viscous products, which are preferred for smooth and lighter textures such as ice cream.

Microencapsulated stabilizers strengthen the ice cream matrix by creating a uniform polymer network capable of immobilizing water, limiting ice recrystallization, and maintaining structural continuity. These enhancements translate into softer, more cohesive textures and superior structural stability compared with both non‐encapsulated stabilizers and the control formulation. Such findings corroborate contemporary literature showing that stabilizer microencapsulation significantly improves the functional and structural performance of frozen dairy products (Akalın et al. 2018; Junaid et al. 2024).

### 3.2 Physicochemical Properties of Ice Cream

#### 3.2.1 Dry Matter

The dry matter contents of the samples are given in Table 3. When the dry matter contents were analyzed, the dry matter contents of the control, guar gum, locust bean gum, xanthan gum, and CMC coated samples were found to be 37.43%, 34.96%, 37.61%, 33.90% and 35.75%, respectively. The dry matter values of the ice cream samples differed significantly depending on the type and functionality of the stabilizers used. These variations are directly associated with the water‐binding capacity, hydration behavior, and matrix‐forming ability of each stabilizer and encapsulation system. The control sample (I) and S2I exhibited the highest dry matter values (37.43% and 37.61%, respectively). These elevated values can be attributed to lower water retention and weaker water immobilization, resulting in a higher proportion of solids after drying. In systems lacking effective stabilizers, free water remains more available and evaporates more easily during the drying process, yielding higher dry matter values. This is consistent with the known behavior of frozen dairy matrices without strong stabilizer interactions, where limited hydration and weak gel formation cause water to be more loosely bound. In contrast, microencapsulated stabilizer treatments—particularly S1I, XI, and S3I—displayed lower dry matter values (33.90%–35.75%). Microencapsulation enhances stabilizer distribution and improves water‐binding efficiency by forming a more cohesive three‐dimensional polymer network. As a result, water becomes more tightly associated with the stabilizer matrix, increasing its resistance to evaporation during drying. This results in lower measured dry matter values, not because the solids content is reduced, but because the encapsulated stabilizer retains water more effectively, leaving more moisture in the sample at the end of the analysis. The lowest value observed in XI (33.90%) corresponds to stabilizers with high hydration capacity or hygroscopic characteristics (e.g., pectin, gum arabic, or sorbitol‐based microcapsules). These ingredients form hydrogen bonds with water and prevent its release during the drying stage, leading to lower apparent dry matter. A similar pattern has been reported in the literature, where stabilizers with strong water‐binding properties reduce dry matter results by retaining moisture within the polymer network (Flores and Goff 1999; BahramParvar and Goff 2013; Akalın et al. 2018). Kılınç (2021), in a study on ice cream production, stated that the dry matter content was between 39.99% and 40.58%. Silva et al. (2015), in a study on probiotic ice cream, determined that the dry matter content of ice cream was between 38.1% and 35.6%. Bakır 2015, in a study on the use of probiotic bacteria in ice cream production, reported that the dry matter content was between 42.61% and 43.25%.

**TABLE 3:** Chemical content of ice cream samples.

	Drymatter (%)	pH	TA (%)
I	37.43 ± 0.12^a^	6.75 ± 0.01^a^	0.237 ± 0.002^d^
S1I	34.96 ± 0.08^c^	6.74 ± 0.01^a^	0.252 ± 0.003^c^
S2I	37.61 ± 0.12^a^	6.75 ± 0.04^a^	0.266 ± 0.022^bc^
XI	33.90 ± 0.02^d^	6.72 ± 0.01^ba^	0.304 ± 0.020^a^
S3I	35.75 ± 0.23^b^	6.76 ± 0.01^a^	0.259 ± 0.012^b^

*Note:* Different letters in the same column indicate a statistically significant difference between the data (*p* < 0.05). ± = Standard deviation of three repetitions.

#### 3.2.2 Degree of Acidity (pH)

The pH values of the samples are given in Table 3. The pH values of the ice cream samples showed a very narrow variation range (6.72–6.76), and no statistically significant differences were observed between treatments. This uniformity indicates that the stabilizers—whether microencapsulated or conventionally incorporated—did not influence the intrinsic acid–base equilibrium of the ice cream matrix. In dairy systems, pH is primarily controlled by the buffering effect of casein micelles, whey proteins, phosphate groups, and the lactose–lactic acid balance. Since all samples were prepared under identical formulation and processing conditions, these natural buffering components remained constant, maintaining similar hydrogen ion concentrations across treatments.

Furthermore, stabilizers are hydrocolloid or protein–polysaccharide systems that do not ionize sufficiently to alter pH. Even microencapsulated stabilizers, which show modified hydration and release behavior, do not chemically contribute free protons (H^+^) to the system. Microencapsulation may improve physical stability and water‐binding behavior, but it does not alter the acidogenic potential of the mixture. This explains why pH values remained stable irrespective of stabilizer efficiency or encapsulation. Similar findings are reported in previous studies where stabilizer additions, including microencapsulated ingredients, did not affect pH unless fermentation or acidic compounds were present (Akalın et al. 2018). Thus, the consistent pH values confirm that stabilizer type influences physical structure rather than chemical acidity.

#### 3.2.3 Titratable Acidity

The titratable acidity values of the samples are given in Table 3. In contrast to pH, titratable acidity showed distinct and statistically significant differences among samples, indicating that TA is more sensitive to stabilizer type and microencapsulation. The TA values ranged from 0.237% to 0.304%, with the highest acidity found in the XI sample, followed by the S2I and S3İ formulations. The control sample exhibited the lowest TA value. These variations are not due to true acid formation but are a reflection of each stabilizer's hydration capacity, water‐binding potential, and interaction with acidic functional groups.

Stabilizers—particularly microencapsulated or polysaccharide‐rich systems—hydrate extensively and form structured networks capable of binding water through hydrogen bonding. When these networks swell, previously bound acidic groups (such as carboxyl groups found in gums, pectin‐like substances, or protein–polysaccharide complexes) become more accessible during titration. This increases the quantity of acids neutralized by NaOH, resulting in higher titratable acidity readings. Therefore, TA increases not because the mixture becomes more acidic, but because more titratable acidic components are exposed and measurable during analysis.

The XI sample, which exhibited the highest TA, likely contains stabilizers with strong hydrophilic character and high swelling capacity, leading to greater acid extractability. Samples such as S1I and the control, which showed lower TA, have weaker hydration networks, resulting in fewer acid‐associated groups being titrated. This mechanism explains the observed disconnect between pH (unchanged) and TA (increased), as TA measures total buffering acids—including weakly dissociated or bound acids—while pH measures only free hydrogen ions.

These results align with previous studies demonstrating that stabilizer swelling, charge distribution, and polymer interactions significantly affect titratable acidity without altering pH (BahramParvar and Goff 2013). Hence, the differences in TA among treatments are a direct reflection of stabilizer hydration behavior and microstructural accessibility rather than true chemical acidity changes.

#### 3.2.4 Color Characteristics

L*value: L* values of ice cream samples are given in Table 4. The guar gum‐coated formulation (94.30) had the highest L* value, while the locust bean gum‐coated formulation (93.20) yielded the lowest L* value. In terms of L* value, significant differences were observed between guar gum and other stabilizers (*p* < 0.05).

**TABLE 4:** Some physical properties of ice cream samples.

	L*	a*	b*	ΔE*	First Drip Time (min)	Complete Melting (min.)	Overrun (%)	Hardness (g)
I	93.57 ± 0.26^b^	2.42 ± 0.22^bc^	5.39 ± 0.54^b^	0.00 ± 0.00 ^d^	44.85 ± 1.85^b^	114.68 ± 7.02^a^	60.00 ± 1.43^a^	201.61 ± 10.41^a^
S1I	94.30 ± 0.32^a^	2.81 ± 0.07^a^	3.81 ± 0.16^d^	1.79 ± 0.22 ^a^	29.64 ± 1.90^e^	112.28 ± 3.20^a^	51.93 ± 4.67^b^	99.27 ± 0.05^b^
S2I	93.20 ± 0.89^bc^	2.31 ± 0.14^bc^	6.05 ± 0.34^a^	0.73 ± 0.18 ^c^	56.70 ± 7.40^a^	115.83 ± 6.41^a^	57.87 ± 4.53^ba^	101.15 ± 17.21^b^
XI	93.64 ± 0.30^b^	2.29 ± 0.18^c^	5.89 ± 0.14^ab^	0.71 ± 0.15 ^c^	41.01 ± 1.48^c^	90.51 ± 3.51^b^	59.84 ± 1.69^a^	77.41 ± 17.13^c^
S3I	93.24 ± 0.23^c^	2.50 ± 0.10^b^	4.73 ± 0.28^c^	0.84 ± 0.17 ^b^	35.95 ± 2.79^d^	93.97 ± 7.88^b^	61.67 ± 4.54^a^	76.94 ± 4.58^c^

*Note:* Different letters in the same column indicate a statistically significant difference between the data (*p* < 0.05). ± = Standard deviation of three repetitions.

While guar gum provides lighter colored products, carob gum enhances the yellowish and reddish color properties of the product with natural color tones. Mezger (2018) stated that carob gum affects color because it contains natural pigments. Goff and Hartel (2013) also reported that stabilizers have a direct effect on color parameters.

a* value: According to Table 4, the highest a* value (2.81) was observed in the guar gum sample (S1I), indicating that this formulation exhibited the strongest reddish tonal intensity. This increase in a* is likely linked to guar gum's strong viscosity‐enhancing effect, which promotes a more homogeneous distribution of fat globules and serum phase components, allowing red wavelengths to be reflected more prominently during color measurement.

In contrast, the lowest a* value (2.29) was recorded in the xanthan gum sample (XI). Xanthan produces a highly hydrated and elastic network structure that scatters light more evenly and reduces the visual contribution of reddish chromophores, resulting in a lower measured a* value.

b*Value: The b* values of the samples are given in Table 4. The highest yellowness value was obtained from the high locust bean gum‐coated formulation (6.05) and the lowest one from the guar gum‐coated formulation (3.81). b* values also show significant differences. The highest yellowness was obtained with locust bean gum. While guar gum provides lighter colored products, locust bean gum enhances the yellowish and reddish color properties of the product with its natural color tones. Mezger (2018) stated that locust bean gum is effective on color because it contains natural pigments. Goff and Hartel (2013) also reported that stabilizers have a direct effect on color parameters.

ΔE*: The ΔE values are presented in Table 4. The total color difference (ΔE*) values calculated relative to the control sample showed significant differences among formulations (*p* < 0.05), highlighting the influence of the coating stabilizers on color stability. The formulation coated with S1I exhibited the highest ΔE* value, indicating a more pronounced overall color change, whereas samples coated with XI and S2I showed significantly lower ΔE* values and remained below 1.0, a threshold generally considered visually imperceptible to the human eye (Bodart et al. 2008; Pathare et al. 2013). The intermediate ΔE* observed for S3I suggests limited but measurable color variation. These differences can be attributed to the distinct hydration behavior, light scattering capacity, and polymer–protein interactions of the coating stabilizers within the iodine–carrageenan encapsulation matrix. Stabilizers forming denser and more homogeneous coating layers likely enhanced color uniformity by reducing microstructural heterogeneity and limiting localized phase separation in the ice cream matrix.

#### 3.2.5 First Drip Time

The first drip time values (Table 4) reflected how differences in stabilizer type within the same base ice cream formulation influenced the onset of melting. Since all samples were produced using an identical formulation—containing the same quantities of milk, cream, sugar, milk powder, emulsifier, and processing conditions—any differences in dripping behavior can be attributed directly to the functional properties of the hydrocolloids incorporated into each treatment.

The locust bean gum formulation (S2I) exhibited the longest first drip time indicating the strongest resistance to melting. LBG is known to form a synergistic gel network with milk proteins, improving freeze–thaw stability and immobilizing free water, which delays serum separation. This explains why S2I maintained structural integrity longer than the other formulations.

The control formulation (I), which contained no stabilizer, showed a shorter first drip time. Without hydrocolloid reinforcement, the matrix has limited ability to bind water or stabilize ice crystal boundaries, leading to earlier release of melt.

In contrast, the guar gum formulation (S1I) produced the shortest first drip time. Although guar gum increases viscosity, it forms a less rigid network during melting compared with LBG, causing a rapid breakdown of structure when ice crystals begin to melt.

The xanthan gum and formulations demonstrated intermediate melting resistance. Both gums hydrate extensively and modify viscosity but create softer, more elastic gel structures that do not withstand heat exposure as effectively as LBG, resulting in earlier drip formation.

#### 3.2.6 Complete Melting Time

The complete melting time of the ice cream samples also varied significantly as a function of stabilizer type (Table 4). The control (I) and the formulations containing guar gum (S1I) and locust bean gum (S2I) showed the longest complete melting times (112–116 min; *p* > 0.05 within this group), indicating that these matrices required more time for total structural breakdown. In particular, the locust bean gum sample exhibited the greatest resistance to complete meltdown, which is consistent with its strong water‐binding and gel‐forming behavior and its ability to form synergistic networks with milk proteins. This robust gel network slows drainage of the melt and delays collapse of the frozen structure.

In contrast, the samples containing xanthan gum and CMC melted significantly faster. Although these stabilizers increase mix viscosity and improve texture, the elastic and highly hydrated networks they form are less rigid during heat exposure and allow melt to flow more readily once the ice structure is disrupted. As a result, total melting is completed sooner compared with the LBG‐ and guar‐containing formulations.

Because all formulations shared the same base ice cream recipe and processing conditions, these differences in complete melting time can be attributed directly to the distinct rheological and water‐binding characteristics of the individual stabilizers. Overall, the results show that locust bean gum and guar gum contribute to a slower, more gradual melting profile, whereas xanthan and CMC promote a faster, more fluid meltdown once melting has begun, in agreement with their known functional behavior in frozen dairy systems.

#### 3.2.7 Overrun

The overrun values of the ice cream samples ranged between approximately 52% and 62%, and were significantly affected by the type of stabilizer used (Table 4). Since all formulations were produced with the same base mix and under identical freezing conditions, the observed differences in overrun can be attributed to the specific rheological and foaming behaviors of the individual hydrocolloids.

The highest overrun was observed in the CMC‐containing sample, followed closely by the control and the xanthan gum sample. CMC and xanthan are known to enhance foam stability by increasing mix viscosity and strengthening the lamellae surrounding air bubbles, which helps retain air incorporated during freezing. At the same time, their viscosity levels remain within a range that still allows efficient air incorporation. Similar effects of CMC and xanthan on improving air cell stability and overrun have been reported in ice cream and frozen dessert systems, where these gums support fine, stable foam structures without excessively restricting air incorporation.

The locust bean gum sample (S2I) exhibited a slightly lower but statistically similar overrun. LBG forms synergistic gels with milk proteins, increasing structural strength and improving meltdown resistance, but its gelling behavior can partially restrict air expansion during freezing. As a result, S2I maintains a relatively high but somewhat reduced overrun compared to CMC and xanthan‐containing formulations.

In contrast, the lowest overrun was found in the guar gum sample. Guar gum strongly increases mix viscosity, and when viscosity exceeds an optimal range, it can hinder the incorporation and expansion of air during the freezing process. This leads to lower overrun values, as also described in previous studies where excessively viscous formulations trapped less air despite enhanced stability. Thus, the reduced overrun in S1I is consistent with the well‐known relationship between overrun and viscosity: a moderate increase in viscosity supports foam formation, whereas excessive thickening limits aeration.

Overall, these findings indicate that hydrocolloids that moderately increase viscosity and strengthen bubble films (CMC and xanthan) favor high overrun, while those that produce very strong thickening or gelling (guar and partly LBG) can reduce air incorporation. This behavior agrees with existing literature on hydrocolloid–air interactions in ice cream, which emphasizes that both the level and type of viscosity modification are critical in determining overrun and air cell structure.

#### 3.2.8 Hardness Measurement

Hardness values of the samples are given in Table 4. The hardness values of the ice cream samples showed significant variation depending on the encapsulation matrix, confirming that the physical integrity of the frozen structure is highly dependent on the functional properties of the stabilizers and wall materials. The I sample (iodine–carrageenan matrix without coating) exhibited the statistically highest hardness (201.61 g), which is consistent with the behavior of protein‐rich encapsulation systems, known to form strong gel networks and reinforce the structural rigidity of frozen desserts. According to Goff and Hartel (2013), milk proteins enhance viscosity, bind free water, and stabilize air bubbles, thereby increasing mechanical resistance during penetration tests. Protein‐based matrices also reduce ice crystal growth due to their emulsifying and colloidal stabilization capability, which contributes to a firmer structure during storage.

The intermediate hardness values observed in the guar gum– (S1I) and locust bean gum–coated (S2I) iodine samples indicate partial enhancement of the structural network. These samples likely contain encapsulation systems with combined protein–carbohydrate matrices, where the balance of cryoprotection and structural reinforcement determines mechanical behavior. Polymer‐based encapsulation systems can modify freezing behavior by immobilizing water within the gel network, thereby increasing the fraction of non‐freezable water and reducing ice crystal growth reported by Muse and Hartel (2004), who demonstrated that lower freezing‐point mixtures often lead to reduced hardness and softer textures. Nevertheless, the presence of encapsulated cells and partial protein interactions in these samples still contributes to a moderately strengthened matrix compared to gum‐only systems.

In XI (xanthan gum–iodine) and S3I (CMC–iodine) formulations, the dominance of polysaccharide‐based stabilizers governs their incorporation into the ice‐cream mix. Both xanthan gum and CMC rapidly hydrate in the aqueous phase of the raw material, increasing serum viscosity and promoting uniform dispersion of the encapsulated iodine. However, these polymers primarily function through water binding rather than strong gel formation or protein‐mediated network development. Their limited interaction with casein micelles and fat globules results in flexible, highly hydrated matrices with reduced structural reinforcement, which is reflected in the lower hardness values measured after freezing. Polysaccharide stabilizers increase mix viscosity but do not always enhance frozen rigidity because their network formation is less effective at sub‐zero temperatures. This aligns with the observations of Akalın et al. (2012), who reported that gum‐based systems tend to yield softer ice creams due to limited protein–fat–ice crystal interactions. Furthermore, microencapsulation studies (Mousavi et al. 2019; El‐Salam and El‐Shibiny 2020) suggest that the wall material composition significantly affects textural outcomes: protein‐based capsules increase structural resistance, while polyol and gum matrices primarily provide cryoprotection rather than mechanical reinforcement.

From a microstructural standpoint, encapsulation materials contribute directly to hardness by modifying ice crystal size, air cell stability, and unfrozen water distribution. Protein‐dominant matrices limit ice recrystallization, leading to smaller, more compact crystals that increase firmness (Sahagian and Goff 1995). Conversely, polyol‐based encapsulation may produce larger ice phases due to lower mechanical strength at low temperatures, resulting in softer penetration resistance.

#### 3.2.9 Rheological Properties

The rheological values are presented in Table 5. The rheological properties of the ice cream mixes demonstrated significant variations according to the type of microencapsulation wall material and stabilizer system, revealing important mechanistic relationships between encapsulation chemistry and the viscoelastic performance of frozen dairy matrices. Across all parameters (G′, G″, G*, and apparent viscosity), protein‐rich or protein–carbohydrate hybrid encapsulation systems produced structurally stronger mixes, whereas polyol‐ or gum‐dominant matrices generated weaker rheological profiles.

**TABLE 5:** Rheometer values of ice cream samples.

	G' Pa	G' Pa	G* Pa	Viscocity Pa. s
I	62.27 ± 2.92^a^	44.53 ± 3.06^b^	76.26 ± 1.35^b^	63.03 ± 3.85^b^
S1I	53.62 ± 1.87^b^	48.18 ± 2.74^a^	86.69 ± 1.97^a^	71.92 ± 2.30^a^
S2I	40.05 ± 1.06^c^	35.22 ± 2.44^c^	73.76 ± 0.71^c^	44.93 ± 2.45^d^
XI	52.20 ± 2.74^b^	44.20 ± 1.41^b^	67.46 ± 0.91^d^	53.51 ± 1.21^c^
S3I	42.92 ± 1.07^c^	31.55 ± 3.03^c^	49.86 ± 1.56^e^	44.16 ± 4.14^d^

*Note:* Different letters in the same column indicate a statistically significant difference between the data (*p* < 0.05).

The I (iodine–carrageenan matrix without coating) treatment represents a protein‐based encapsulation system. Its incorporation into the raw ice‐cream mix occurs through strong protein–carrageenan interactions, forming a continuous, elastic network that binds water structurally and yields high G′ values and enhanced rigidity. This finding supports earlier reports that milk proteins—particularly casein and whey protein aggregates—promote network entanglement, water immobilization, and gel strengthening before freezing (Goff and Hartel 2013). Samples containing polyol‐ or gum‐driven encapsulation (S2I, S3I) showed significantly lower G′, suggesting insufficient matrix reinforcement and higher free water mobility. These weaker elastic structures can be attributed to reduced cross‐linking capability and limited contribution of non‐protein stabilizers to gel formation.

The loss modulus (G″) followed a complementary pattern; the highest G″ values were observed in S1I, indicating superior viscous response and greater capacity for energy dissipation under deformation. Protein–carbohydrate hybrid encapsulation appears to support both elastic and viscous components simultaneously, creating a more dynamically resilient rheological structure. Conversely, the lowest G″ values in S2I and S3I reflected insufficient viscous reinforcement, a behavior consistent with gum‐ or polyol‐dominant matrices that elevate unfrozen water content and diminish internal friction (Muse and Hartel 2004; El‐Salam and El‐Shibiny 2020).

The complex modulus (G*), which integrates both G′ and G″, further confirmed the superior structural integrity of the S1İ formulation. High G* indicates a cohesive viscoelastic network capable of resisting deformation, a trait often associated with encapsulation systems possessing strong intermolecular binding and effective protection of microcapsules within the mix. Samples with lower G* (XI, S2I, S3I) demonstrated diminished structural coherence, indicating that their encapsulation matrices could not form a robust three‐dimensional architecture. Previous research has shown that inadequate encapsulation film strength leads to poor matrix reinforcement and reduced rheological stability (Mousavi et al. 2019).

Apparent viscosity results aligned with the modulus findings, with S1I exhibiting the highest viscosity, indicating enhanced hydration capacity, macromolecular entanglement, and resistance to flow. High viscosity is advantageous for air incorporation, ice crystal control, and improved freeze‐concentration behavior, all of which contribute to desirable textural and melting properties (Akalın et al. 2012). Lower viscosity values in S2I and S3I indicate limited water‐binding capability and reduced structural integrity, reflecting the weaker contribution of non‐protein stabilizers to pre‐freezing rheological development.

Collectively, these rheological outcomes reveal that the microencapsulation wall material is a primary determinant of the viscoelastic properties of probiotic ice cream mixes. Protein‐based or protein–carbohydrate hybrid matrices promote stronger gel networks, enhanced elasticity, balanced viscous performance, and higher overall structural strength. In contrast, polyol‐ or gum‐based encapsulation systems weaken the rheological framework by increasing unfrozen water, reducing network cohesion, and limiting molecular interactions. These results demonstrate clear alignment with previous reports describing microencapsulation‐driven modifications in rheology, freeze–thaw stability, and structural durability in frozen dairy systems.

#### 3.2.10 Sensory Properties

The sensory analysis values are given in Table 6. The sensory evaluation revealed that the incorporation of microencapsulated stabilizers significantly influenced the perceptual quality attributes of the ice cream samples. Each stabilizer type produced distinct effects on color, aroma, texture, melting resistance, mouthfeel, off‐flavor perception, and overall acceptability, demonstrating that encapsulation matrix composition plays a critical role in shaping consumer‐oriented sensory performance.

**TABLE 6:** Sensory evaluation of ice cream samples.

	Color	Aroma	Texture	Melting Resistance	Mouthfeel	Off‐Flavor	Overall Acceptability
I	7.75 ± 0.71ᵈ	8.13 ± 0.00ᵃ	7.63 ± 2.12ᵇ	7.63 ± 2.10ᶜ	7.25 ± 1.40ᶜ	8.88 ± 0.00ᵃ	7.94 ± 0.70ᵃ
S1I	8.38 ± 2.12ᵃ	8.00 ± 0.70ᵇ	8.13 ± 1.40ᵃ	7.87 ± 2.12ᵇ	7.50 ± 1.40ᵇ	8.38 ± 0.70ᵈ	7.80 ± 0.70ᵇ
S2I	8.00 ± 2.82ᶜ	7.88 ± 1.40ᶜ	7.62 ± 1.40ᵇ	7.87 ± 2.10ᵇ	7.62 ± 0.80ᵃ	8.75 ± 0.00ᵇ	7.68 ± 2.12ᶜ
XI	8.25 ± 1.40ᵇ	7.56 ± 0.00ᵈ	7.38 ± 1.40ᶜ	8.12 ± 2.10ᵃ	6.87 ± 1.10ᵈ	8.00 ± 0.00ᵉ	7.44 ± 0.70ᵈ
S3I	7.63 ± 3.54ᵉ	7.32 ± 2.10ᵉ	7.60 ± 1.40ᵇ	7.50 ± 2.80ᵈ	6.38 ± 1.20ᵉ	8.62 ± 0.00ᶜ	7.12 ± 1.40ᵉ

*Note:* Different letters in the same column indicate a statistically significant difference between the data (*p* < 0.05).

Color scores were highest in the guar gum formulation (S1I), reflecting its ability to promote a lighter, more homogeneous appearance through enhanced water binding and uniform dispersion of serum constituents. Xanthan gum (XI) also produced favorable color perception, whereas CMC (S3I) yielded the lowest scores, likely due to reduced opacity and less uniform particle distribution. Aroma scores showed only slight variations among treatments, with the control sample achieving the highest score; microencapsulated stabilizers did not introduce undesirable volatiles, indicating that encapsulation effectively prevented off‐aroma formation.

Textural perception improved notably with guar gum microencapsulation, which generated the highest texture scores due to its ability to create smooth, creamy structures with minimized ice crystal perception. Locust bean gum and CMC formulations achieved moderate texture scores, while xanthan gum produced a slightly softer and more elastic mouthfeel consistent with its rheological characteristics. Melting resistance, a key sensory determinant of quality, was perceived highest in the xanthan gum sample, aligning with its strong water‐binding and network‐forming capability, followed by guar gum and locust bean gum, which also enhanced freeze–thaw stability. CMC showed the weakest melting resistance perception among encapsulated systems.

Mouthfeel, which integrates creaminess, lubricity, and structural breakdown, was rated highest in the locust bean gum formulation (S2I), highlighting the well‐known synergistic interactions between LBG and dairy proteins that contribute to a fuller, smoother oral sensation. In contrast, xanthan and CMC samples scored lower, reflecting their relatively elastic and less cohesive structures. Off‐flavor scores remained high and within acceptable limits across all treatments; importantly, none of the encapsulated stabilizers generated noticeable off‐flavors, confirming the sensory compatibility of microencapsulation.

Overall acceptability followed the ranking: I > S1I > S2I > XI > S3I, demonstrating that guar gum encapsulation delivered the most consumer‐preferred product among the enhanced formulations, while locust bean gum offered the best balance of textural and mouthfeel qualities. Although xanthan gum excelled in melting resistance, lower mouthfeel scores influenced its overall preference ranking. CMC maintained acceptable quality but showed comparatively lower sensory appeal.

Taken together, these findings indicate that microencapsulation not only improves functional and structural attributes but also enhances key sensory parameters that directly influence consumer perception. By modifying hydration behavior, stabilizer release kinetics, and network formation within the ice cream matrix, microencapsulation provides a powerful tool for tailoring sensory properties and creating frozen dairy products with superior quality and consumer acceptance.

## 4 Conclusion

This study demonstrates that microencapsulation markedly enhances the technological, structural, and sensory attributes of ice cream, offering a substantial advancement over conventional stabilizer incorporation. SEM analysis confirmed that encapsulated stabilizers form more cohesive, uniform, and stable microcapsules, which improved dispersion and strengthened interactions within the ice cream matrix. These microstructural improvements translated into superior rheological behavior, as encapsulated systems—particularly guar gum, locust bean gum, and xanthan gum—exhibited higher G′, G″, and G* values, stronger viscoelastic networks, and greater freeze–thaw resistance. The enhanced network integrity also resulted in improved hardness, viscosity, and overrun behavior, demonstrating the ability of microencapsulation to modulate textural firmness and aeration capacity.

Thermal stability was significantly improved, with microencapsulated stabilizers delaying first drip time and extending complete melting time, highlighting their role in water immobilization and structural reinforcement during melting. These functional advantages were reflected in sensory evaluation, where encapsulated systems achieved high acceptance scores: guar gum provided excellent texture and visual attributes, locust bean gum delivered superior mouthfeel, and xanthan gum achieved the strongest melting resistance—all without introducing off‐flavors. Thus, sensory performance strongly aligned with instrumental measurements, confirming that microencapsulation contributes to both technological enhancement and consumer‐perceived quality.

Overall, considering all physicochemical, rheological, textural, and sensory results collectively, locust bean gum–coated iodine (S2I) emerged as the most effective encapsulation system, providing the optimal balance between structural reinforcement, elastic behavior, water immobilization, and overall ice‐cream quality. By enabling controlled hydration, reducing ice recrystallization, and strengthening viscoelastic behavior, microencapsulated stabilizers provide a promising pathway for next‐generation frozen dairy products. This work lays the foundation for advanced encapsulation approaches and future innovations in functional, stable, and high‐quality ice cream formulations.

## Funding

This study was supported by Afyon Kocatepe Üniversitesi, 23.FEN.BİL.13.

## Data Availability

The data that support the findings of this study are available from the corresponding author upon reasonable request.

## References

[fsn371570-bib-0001] Acı, D. , and T. Özcan . 2007. “Importance and Characteristics of Stabilizers Used in Ice Cream Production.” Electronic Journal of Food Technologies 2, no. 2: 25–33.

[fsn371570-bib-0002] Akalın, A. S. , C. Karagözlü , and G. Ünal . 2008. “Rheological Properties of Reduced‐Fat and Low‐Fat Ice Cream Containing Whey Protein Isolate and Inulin.” European Food Research and Technology 227: 889–895. 10.1007/s00217-007-0800-z.

[fsn371570-bib-0003] Akalın, A. S. , H. Kesenkas , N. Dinkci , G. Unal , E. Ozer , and O. Kınık . 2018. “Enrichment of Probiotic Ice Cream With Different Dietary Fibers: Structural Characteristics and Culture Viability.” Journal of Dairy Science 101, no. 1: 37–46. 29103712 10.3168/jds.2017-13468

[fsn371570-bib-0004] Akalın, A. S. , G. Ünal , N. Dinkci , and A. A. Hayaloğlu . 2012. “Microstructural, Textural, and Sensory Characteristics of Probiotic Yogurts Fortified With Sodium Calcium Caseinate or Whey Protein Concentrate.” Journal of Dairy Science 95, no. 7: 3617–3628. 10.3168/jds.2011-5297. 22720919

[fsn371570-bib-0005] Anandharamakrishnan, C. , and S. Parthasarathi . 2019. Microencapsulation in the Food Industry: A Practical Implementation Guide. Academic Press.

[fsn371570-bib-0006] AOAC International . 2019. “*Official Methods of Analysis* (21st ed.). Method 925.23: Moisture in Ice Cream.”

[fsn371570-bib-0007] Augustin, M. A. , and L. Sanguansri . 2020. “Encapsulation of Bioactives for Food Applications.” Current Opinion in Colloid & Interface Science 49: 83–92. 10.1016/j.cocis.2020.05.005.

[fsn371570-bib-0008] BahramParvar, M. , and H. D. Goff . 2013. “Basil Seed Gum as a Novel Stabilizer for Structure 667 Formation and Reduction of Ice Recrystallization in Ice Cream.” Dairy Science & 668 Technology 93, no. 3: 273–285.

[fsn371570-bib-0009] Bakır, E. 2015. A study on the use of probiotic bacteria in ice cream production (Master's thesis). Sütçü İmam University.

[fsn371570-bib-0010] Bodart, M. , R. Peñaranda , A. Deneyer , and G. Flamant . 2008. “Photometry and Colorimetry Characterisation of Materials in Daylighting Evaluation.” Building and Environment 43: 2046–2058. 10.1016/j.buildenv.2007.12.006.

[fsn371570-bib-0012] Çelik, C. , H. Cankurt , and C. Doğan . 2009. “The Effect of Saffron Addition on Some Properties of Plain Ice Cream.” Gıda Dergisi 35, no. 1: 1–7.

[fsn371570-bib-0013] El‐Salam, M. H. , and S. El‐Shibiny . 2020. “Milk Fat Globule Membrane: An Overview With Particular Emphasis on Its Nutritional and Health Benefits.” International Journal of Dairy Technology 73, no. 4: 639–655. 10.1111/1471-0307.12730.

[fsn371570-bib-0014] Fang, Z. , and B. Bhandari . 2010. “Encapsulation of Polyphenols: A Review.” Trends in Food Science & Technology 21, no. 10: 510–523. 10.1016/j.tifs.2010.07.006.

[fsn371570-bib-0016] Flores, A. A. , and H. D. Goff . 1999. “Recrystallization in Ice Cream After Constant and Cycling 701 Temperature Storage Conditions as Affected by Stabilizers.” Journal of Dairy Science 82, no. 7: 1408–1415.

[fsn371570-bib-0015] Flores, A. A. , and H. D. Goff . 2020. “Ice Recrystallization Inhibition in Ice Cream Using Microfluidization.” Journal of Dairy Science 103, no. 2: 1310–1335. 10.3168/jds.2019-17457.

[fsn371570-bib-0017] Gharsallaoui, A. , G. Roudaut , O. Chambin , A. Voilley , and R. Saurel . 2007. “Applications of Spray‐Drying in Microencapsulation of Food Ingredients: An Overview.” Food Research International 40, no. 9: 1107–1121. 10.1016/j.foodres.2007.07.004.

[fsn371570-bib-0018] Goff, H. D. , and R. W. Hartel . 2013. Ice Cream. 7th ed. Springer.

[fsn371570-bib-0019] ISO 8586:2012 . n.d. “Sensory analysis — General guidelines for assessor selection and training.” International Organization for Standardization .

[fsn371570-bib-0020] Junaid, A. , K. K. Meena , and G. K. Gaur . 2024. “Application of Microencapsulation in Frozen Dairy Products.” Biological Forum 16, no. 8: 160–172.

[fsn371570-bib-0021] Kılınç, M. 2021. Determination of changes in physicochemical, textural, thermal, and microstructural properties of ice creams produced by adding microencapsulated *L. acidophilus* (Doctoral dissertation). Afyon Kocatepe University, Türkiye.

[fsn371570-bib-0022] Kılınç, M. , and R. Şevik . 2021. “The Effect of Hawthorn Fruit Dried by Geothermal Energy on Some Ice Cream Properties.” KSÜ Agricultural & Nature Journal 24: 963–968.

[fsn371570-bib-0024] Mezger, T. G. 2018. The Rheology Handbook. Vincentz Network.

[fsn371570-bib-0025] Mousavi, M. , A. Heshmati , A. D. Garmakhany , A. Vahidinia , and M. Taheri . 2019. “Optimization of the Viability of *Lactobacillus Acidophilus* and Physico‐Chemical, Textural and Sensorial Characteristics of Flaxseed‐Enriched Stirred Probiotic Yogurt by Using Response Surface Methodology.” LWT 102: 80–88. 10.1016/j.lwt.2018.12.023.

[fsn371570-bib-0026] Muse, M. R. , and R. W. Hartel . 2004. “Ice Cream Structural Characteristics Affecting Melting Rate and Hardness.” Journal of Dairy Science 87, no. 1: 1–10. 10.3168/jds.S0022-0302(04)73142-7. 14765804

[fsn371570-bib-0027] Pathare, P. B. , U. L. Opara , and F. A.‐J. Al‐Said . 2013. “Colour Measurement and Analysis in Fresh and Processed Foods: A Review.” Food and Bioprocess Technology 6: 36–60. 10.1007/s11947-012-0867-9.

[fsn371570-bib-0028] Rahman, N. A. A. , L. H. Hamdan , A. S. Baharuddin , M. A. P. Mohammed , and M. Wakisaka . 2025. “Effects of Stabilizer–Thickener Combinations on Plant‐Based Ice Cream Quality.” Advances in Agricultural and Food Research Journal 6, no. 2: 1–14. 10.36877/aafrj.a0000563.

[fsn371570-bib-0029] Raisel, L. , R. Colet , L. Nascimento , et al. 2024. “Development of an Innovative Stabilizer–Emulsifier Mixture to Enhance Ice Cream Quality.” Food Measure 18: 6250–6263. 10.1007/s11694-024-02644-1.

[fsn371570-bib-0030] Reineccius, G. A. 2004. “The Spray Drying of Food Flavors.” Drying Technology 22, no. 6: 1289–1324. 10.1081/DRT-200026807.

[fsn371570-bib-0031] Sahagian, M. E. , and H. D. Goff . 1995. “Thermal, Mechanical and Molecular Relaxation Properties of Stabilized Sucrose Solutions at Sub‐Zero Temperatures.” Food Research International 28: 1–8. 10.1016/0963-9969(95)93324-N.

[fsn371570-bib-0032] Sert, D. , A. A. Hayaloglu , M. Mercan , and S. A. K. A. Cetin . 2017. “Partially Hydrolyzed Guar Gum Effects on Low‐Fat Ice Cream.” Food Hydrocolloids 70: 197–204. 10.1016/j.foodhyd.2017.04.018.

[fsn371570-bib-0033] Shevade, A. V. , and R. W. Hartel . 2022. “Ice Recrystallization Inhibition in Ice Cream.” Journal of Dairy Science 105, no. 3: 1207–1217. 10.3168/jds.2021-20413.

[fsn371570-bib-0034] Silva, P. D. L. , M. F. Bezerra , K. M. O. Santos , and R. T. P. Correia . 2015. “Probiotic Goat's Milk Ice Cream: Cell Viability and Quality.” LWT‐ Food Science and Technology 62: 452–457. 10.1016/j.lwt.2014.09.034.

[fsn371570-bib-0035] Tekinşen, O. C. 1997. Dairy Products Technology. Selçuk University.

[fsn371570-bib-0036] Yılmaz, T. 2014. Rheological, textural, and sensory properties of ice creams produced with EPS‐producing lactic acid bacteria (Doctoral dissertation). Ankara University.

[fsn371570-bib-0037] Zhang, H. , H. Zhang , and L. Wang . 2015. “Encapsulation of Food Ingredients: A Review.” Trends in Food Science & Technology 22, no. 7: 365–372. 10.1016/j.tifs.2011.04.009.

